# Life Comparative Analysis of Energy Consumption and CO_2_ Emissions of Different Building Structural Frame Types

**DOI:** 10.1155/2013/175702

**Published:** 2013-10-09

**Authors:** Sangyong Kim, Joon-Ho Moon, Yoonseok Shin, Gwang-Hee Kim, Deok-Seok Seo

**Affiliations:** ^1^School of Construction Management and Engineering, University of Reading, Reading RG6 6AW, UK; ^2^Department of Plant & Architectural Engineering, Kyonggi University, Gwanggyosan-ro, Yeongtong-gu, Suwon-si, Gyeonggi-do 443-760, Republic of Korea; ^3^Department of Architectural Engineering, Halla University, Wonju-si 220-712, Republic of Korea

## Abstract

The objective of this research is to quantitatively measure and compare the environmental load and construction cost of different structural frame types. Construction cost also accounts for the costs of CO_2_ emissions of input materials. The choice of structural frame type is a major consideration in construction, as this element represents about 33% of total building construction costs. In this research, four constructed buildings were analyzed, with these having either reinforced concrete (RC) or steel (S) structures. An input-output framework analysis was used to measure energy consumption and CO_2_ emissions of input materials for each structural frame type. In addition, the CO_2_ emissions cost was measured using the trading price of CO_2_ emissions on the International Commodity Exchange. This research revealed that both energy consumption and CO_2_ emissions were, on average, 26% lower with the RC structure than with the S structure, and the construction costs (including the CO_2_ emissions cost) of the RC structure were about 9.8% lower, compared to the S structure. This research provides insights through which the construction industry will be able to respond to the carbon market, which is expected to continue to grow in the future.

## 1. Introduction

The greenhouse gas (GHG) emissions reduction policy, driven by European Union (EU) members, took effect in 2008 and has been implemented for the past 5 years [1]. This policy is a result of the adoption of the Kyoto Protocol by the United Nations Framework Convention on Climate Change (UNFCC) in 1997. A Post-Kyoto Regime is now emerging, including within it countries classified as developing (such as Korea and Mexico), and within which enforcement of relevant provisions will commence after 2013 [2]. Aside from participating on a voluntary basis in the UNFCCC, developing countries are required to actively respond to the carbon market. In addition, the parties to the UNFCCC are activating carbon trading as a new market-based growth engine to cost-effectively manage their reduction commitments. Furthermore, with the rise of green protectionism, which is the imposition of trade sanctions on countries not participating in the UNFCCC [2], participation in the latter becomes necessary to sustain economic growth, particularly for countries like Korea that have a strong dependence on trade. According to Korea's Ministry of Environment, in 2009, Korea voluntarily submitted to the UN a forecast target of 30% reduction in GHG emissions by 2020; the country also issued advance notice of a GHG emissions trading scheme [3]. Furthermore, all sectors, including the construction industry, have been pursuing research on CO_2_ reduction technology and energy efficiency. The reduction of CO_2_ is particularly essential in the construction industry, a large-scale consumption field, which accounts for more than 40% of natural resource consumption, 30% of energy consumption, and 30% of CO_2_ emissions, and which is in turn based on the steel, petrochemical, and cement industries, which are all major sources of CO_2_ emissions [4].

Few studies have been conducted on the economic aspects of energy consumption and CO_2_ emissions, to enable companies to actively and economically respond to environmental policies, such as those resulting from the UNFCCC and emissions trading schemes. If a country or a company does not actively respond to the UNFCCC provisions, it risks having to pay a large CO_2_ emissions cost. This damages the image of a country or company and eliminates its competitiveness in overseas exports. Notwithstanding, awareness of environmental issues and efforts to resolve them in the private sector have been insufficient. In a survey by Korea's Ministry of Environment, large companies that consume a significant amount of energy were questioned in terms of the necessity of a new economic order due to climate change. However, only 30.8% of respondents replied that this was certainly necessary. Only 6.0% of participating companies were carrying out a GHG reduction program at the time [5].

There are studies [5, 6] that show the energy consumption and CO_2_ emissions of different constructional methods and building materials. Kim et al. [7] quantitatively assess environmental load through calculating energy consumption and CO_2_ emission of building material in apartment construction. Lee et al. [8] compare construction costs, including CO_2_ emission costs of masonry wall. Their study found that CO_2_ emission costs were highest for fire brick walls, followed by concrete brick walls. Some studies [9–15] have been explicitly dedicated to life-cycle analysis (LCA) of material and component combinations. Cole and Kernan [9] examined total life-cycle energy use, including initial embodied energy, recurring embodied energy (associated with maintenance and repair), and operating energy. Wu et al. [11] present a method, which categorizes environmental impacts, using materials' environmental profiles to assess their environmental impacts based on the LCA framework. Gustavsson and Sathre [13] study changes in energy and CO_2_ balances, caused by the variation of key parameters in the manufacture and use of materials which make up a wood and a concrete-framed building. Other research on the LCA of the whole process of construction is ongoing. This includes (i) research that analyzes initial, recurring, demolition, and operating embodied energy of proposed timber, steel, and concrete buildings, by dividing work phases as follows: site work, structure, envelope, finishes, services, and construction of the building [16]; (ii) research that quantifies the total amount of energy consumption and CO_2_ emissions caused by construction, operation, maintenance [17], and renovation of office buildings in Japan; (iii) research to estimate CO_2_ emissions in the life-cycle of residential buildings [18]; (iv) research to measure the life-cycle energy and environmental performance of a new university [19]; (v) research to estimate the environmental load of wood and steel reinforced concrete housing construction [20]; and (vi) a detailed analysis of environmental load for each of the three types of residential buildings in Beijing across their whole life-cycle, focusing on energy consumption and CO_2_ emissions during the phases of embodied materials, construction, operation, and disposal [21].

Although many studies are being conducted, and Korea has been required to cope with a carbon market since its participation in the UNFCCC, its research on CO_2_ emissions costs, as an element of construction costs, has been insufficient. Therefore, this study aims to measure and compare energy consumption, CO_2_ emissions, and construction costs for different structural frameworks, with this analysis also including the CO_2_ emissions cost of input materials. This research has thus included an analysis of the environmental loads of six office buildings with different building structural frame types during the construction stage, using I/O analysis [6], measurement of environmental loads [22], estimation of energy consumption and CO_2_ emissions [7], and qualitative analysis of energy consumption and CO_2_ emissions.

As per this outline of the structure of the research, this paper first considers previous research and functional unit estimation approaches for energy consumption and CO_2_ emissions. Next, energy consumption, CO_2_ emissions, and CO_2_ emission costs of input materials of different structural frame types will be analyzed and compared. Finally, key conclusions will be drawn.

## 2. Research Methodology

This research has been conducted using I/O analysis, based on an interindustry relation table. The I/O table published by the Bank of Korea in 2010 was adapted for this research. By comparing the characteristics of estimation approaches [23], it was found that I/O analysis was more efficient than any other approach in terms of operating hours, scope, and cost. 

**Figure 1:**
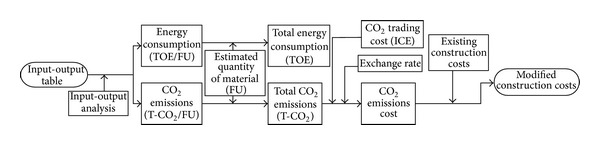
Flowchart for estimation of energy consumption and CO_2_ emissions and modified construction costs.

An outline of the research is presented in Figure 1. First, energy consumption and CO_2_ emissions were estimated by functional unit, using I/O analysis. Functional unit approaches for estimating energy consumption and CO_2_ emissions costs across the life-cycle are divided into three: (i) I/O analysis, directly employing the I/O table; (ii) survey-based approaches that directly track and investigate the same; and (iii) hybrids that combine both approaches [24, 25]. The units of energy consumption and CO_2_ emissions used were TOE (ton of oil equivalent, 107 kcal) and T-CO_2_, respectively. Second, total energy consumption and total CO_2_ emissions of input materials were estimated for each structural frame type. In this research, input materials of four constructed buildings were analyzed, comprising two short and two tall buildings with RC and S structures. Finally, construction costs were estimated, taking into account the CO_2_ emissions cost. CO_2_ emissions cost was measured using the trading price of CO_2_ emissions on the International Commodity Exchange (ICE) and applying the basic rate of exchange of the Korea Exchange Bank. Additionally, modified construction costs were estimated, including estimated CO_2_ emissions cost, and existing construction cost, consisting of material costs, labor costs, and expenses.

## 3. Comparison of Energy Consumption, CO_2_ Emissions, and CO_2_ Emission Costs

### 3.1. Description of Cases

Table 1 provides an outline of the different case study buildings and summarizes the quantity of input materials used for each type of structural frame. All case study buildings are located in Seoul, and all are office buildings. Cases A and B have similar gross floor area (GFA), as do cases C and D. Analyzed input materials include cement, sand, gravel, shape steel, steel sheet, and steel plate.

### 3.2. Estimation of Energy Consumption and CO_2_ Emissions

Table 2 shows estimates for consumed energy and CO_2_ emissions involved in the production of input materials for each structural frame type, as per the I/O analysis.

The energy consumption by unit area (m^2^) was estimated at an average of 0.10 TOE/m^2^ for the RC structure and 0.14 TOE/m^2^ for the S structure. The analysis of energy consumption by input materials showed that steel manufacture has higher energy consumption than the manufacture of any other material used in structural frameworks. 

In the RC structure, “rebar” accounted for about 74% (case A) and 76% (case C) of the total energy consumption. In contrast with the S structure, however, “shaped steel” only accounted for about 4% (A) and 1.9% (C) of total energy consumption. For the S structure, the sum of section “rebar” and “shaped steel” accounted for about 37% and 39% (B) and 26% and 54% (D), respectively.

CO_2_ emissions by unit area (m^2^) were estimated at an average of 0.41 T-CO_2_/m^2^ and 0.55 T-CO_2_/m^2^ for the RC structure and the S structure, respectively. The analysis of CO_2_ emissions by input materials concluded that rebar for the RC structure accounted for the highest portion of total CO_2_ emissions, at 75% (A) and 77% (C), whereas the sum of section shaped steel and rebar for the S structure accounted for 76% (B) and 80% (D). In contrast, sand and gravel emitted less CO_2_ than any other construction material.

### 3.3. Construction Costs Including CO_2_ Emissions Cost

As shown in Table 3, the cost of CO_2_ emissions by unit area (m^2^) was estimated at an average of 8,669 KRW/m^2^ (7.6 USD/m^2^) for the RC structure and an average of 11,759 KRW/m^2^ (10.31 USD/m^2^) for the S structure, with both of these accounting for about 4%–5.3% of existing construction costs. Additionally, by analyzing the rate of increase for modified construction costs (including CO_2_ emissions cost against existing construction costs for different structural frame types), it was noted that the RC structure increased to an average of 4.17% and the S structure to an average of 5.15%. Existing construction costs per unit area (m^2^) were estimated at an average of 208,183 KRW/m^2^ (182.62 USD/m^2^) for the RC structure and at an average of 228,662 KRW/m^2^ (200.58 USD/m^2^) for the S structure. As for modified construction costs, the RC structure and the S structure were estimated at 216,852 KRW/m^2^ (190.22 USD/m^2^) and 240,421 KRW/m^2^ (210.9 USD/m^2^), respectively.

### 3.4. Results and Discussion

The RC structure showed lower energy consumption and CO_2_ emissions than the S structure. Results for the RC structure indicated about 29% less energy consumption and 26% lower CO_2_ emissions per unit area (m^2^) than the S structure. A large portion of emissions is attributable to the steel manufacturing process.

The CO_2_ emissions cost by unit area (m^2^) of the S structure was 26% higher than that of the RC structure. Additionally, by comparing the modified construction cost, it was found that the RC structure reduces costs by about 10% compared to the S structure. The RC structure reduces costs by about 9% compared to existing construction costs, but the cost-saving effect becomes magnified when considering the high CO_2_ emission cost of the S structure.

Therefore, when selecting a structural frame type, it is advantageous to select the RC structure over the S structure, in terms of reducing energy consumption, CO_2_ emissions, and CO_2_ emissions cost. Furthermore, it is more advantageous to select constructional methods or materials that minimize the use of steel, provided that they meet required design conditions.

It is reasonable to consider the CO_2_ emissions cost as part of construction costs, because this cost has an influence on whether it is possible to respond cost-effectively to the UNFCCC and GHG emissions trading scheme. It is understood that a quantitative comparison of CO_2_ emissions and CO_2_ emissions costs for different constructional methods may induce construction companies to select a construction method that will lead to lower levels of energy consumption, CO_2_ emissions, and CO_2_ emissions costs.

## 4. Conclusion

For the construction industry to respond to the UNFCCC and GHG emissions trading scheme in a cost-effective manner, this research has qualitatively estimated and compared energy consumption, CO_2_ emissions, and CO_2_ emissions costs for construction materials used in different structural frame types. 

The values of energy consumption and CO_2_ emissions obtained for buildings analyzed in this study differ from values obtained in other recent studies, primarily due to the use of more current energy consumption and CO_2_ emissions data. Previously published studies on energy consumption and CO_2_ emissions provide important results based on the use of different construction materials, such as masonry wall type (e.g., brick wall, concrete brick wall, and fire brick wall). However, the scope of this study is limited to input materials used in different structural frameworks. Therefore, it is difficult to interpret and compare the studies in any detail, because of the lack of definition of what was included within estimates of total energy consumption and CO_2_ emissions.

Through case studies of buildings constructed with RC structures and S structures, this study has determined that RC structures have the following advantages: (1) reduced energy consumption and CO_2_ emissions; and (2) reduced construction costs, when taking into account the CO_2_ emissions cost. Therefore, in the selection of a structural frame type, it is advantageous to select an RC structure over an S structure, in order to reduce energy consumption, CO_2_ emissions, and CO_2_ emissions costs. 

This resulted from differences in required building materials according to the building structure. It is understood that a quantitative comparison of CO_2_ emissions and CO_2_ emissions costs for different constructional methods may induce construction companies to select a construction method that will lead to lower levels of energy consumption, CO_2_ emissions, and CO_2_ emissions costs. Through this comparison, the construction industry will be able to react to the carbon market, which is expected to continue to grow. In future, it will be necessary to study the emission costs of various constructional methods, using LCA, and thus taking into account the production of construction materials, as well as construction, maintenance, and demolition phases. 

## Figures and Tables

**Table 1:** Case study buildings: summary of input materials.

Section	A	B	C	D

Structure	RC	S	RC	S

Storeys	F15 + B5	F14 + B6	F12 + B4	F14 + B5

Gross floor area				
Aboveground (m^2^)	12,566.37	11,930.45	30,785.75	31,993.80
Underground (m^2^)	6,295.24	7,079.33	20,173.86	19,082.83
Total (m^2^)	**18,861.61**	**19,027.78**	**50,959.81**	**51,076.63**

Quantity of input materials				
Cement (Ton)	4726.36	4396.18	12,643.88	7,300.60
Sand (m^3^)	7069.05	6575.22	18,911.00	10,919.25
Gravel (m^3^)	8123.68	7556.17	21,732.34	12,548.29
Rebar (Ton)	1675.36	1125.61	4382.00	2,076.15
Shape steel (Ton)	75.97	979.89	91.19	3,531.00
Galvanized steel sheet (m^2^)	—	13850.00	—	31,994.00
Steel plate (Ton)	9.44	91.77	7.30	346.84

**Table 2:** Energy consumption and CO_2_ emissions, as per I/O table.

Materials	Energy consumptions					CO_2_ emissions				
	Unit	A	B	C	D	Unit	A	B	C	D
Cement	TOE	401.74	373.68	1,074.89	620.64	T-CO_2_	1,521.89	1,415.57	4,068.35	2,349.07
Sand		7.07	6.58	15.61	9.01		21.21	19.73	50.23	29.00
Gravel		8.12	7.56	17.12	9.88		24.37	22.67	55.08	31.81
Rebar		1,440.81	968.03	3,768.03	1,785.25		5,806.80	3,901.36	15,189.01	7,196.40
Shape steel		78.78	1,016.15	94.56	3,661.69		316.49	4,082.22	379.88	14,709.70
Galvanized steel sheet		—	152.35	—	367.76		—	623.25	—	1,434.36
Steel plate		10.12	98.38	7.82	371.90		40.79	396.54	31.52	1,498.73
Total		**1,946.65**	**2,622.70**	**4,978.03**	**6,826.15**		**7,731.55**	**10,461.34**	**19,774.08**	**27,249.08**
Per area	TOE/m^2^	0.10	0.14	0.10	0.13	T-CO_2_/m^2^	0.41	0.55	0.39	0.53

**Table 3:** Construction costs, including CO_2_ emissions costs.

Construction costs			A	B	C	D
Existing	Subtotal (*α*)	(Million won)	4,138.36	4,576.31	10,037.09	11,074.34
	Per area	(Million won/m^2^)	0.2194	0.2405	0.1970	0.2168

CO_2_ emissions	Subtotal (*β*)	(Million won)	168.05	227.01	429.47	591.81
	Per area	(Million won/m^2^)	0.0089	0.0119	0.0084	0.0116

Modified	Total (*α* + *β*)	(Million won)	4,306.42	4,803.32	10,466.55	11,666.15
	Per area	(Million won/m^2^)	0.2283	0.2524	0.2054	0.2284

Rate of increase (*β*/*α*)			4.06	4.96	4.28	5.34
